# RapidArc for centrally recurrent cervical cancer in the vaginal cuff following primary surgical therapy: a case report

**DOI:** 10.1186/s12957-016-0770-3

**Published:** 2016-01-22

**Authors:** I. Lalya, A. Maghous, E. Marnouche, N. Zaghba, K. Andaloussi, M. Elmarjany, K. Hadadi, H. Sifat, H. Mansouri

**Affiliations:** 1 Department of Radiotherapy, Mohamed V Military Teaching Hospital, Rabat, Morocco; 2 Resident of Radiation Oncology, National Institute of Oncology, Rabat, Morocco

**Keywords:** RapidArc radiotherapy, Recurrent cervical cancer

## Abstract

**Background:**

Pelvic recurrences of cervical cancer after primary surgical treatment can be potentially cured with radical hysterectomy or chemoradiation therapy. Combined radio-chemotherapy is believed to improve results compared to other option. Currently, RapidArc radiotherapy is considered an excellent technological advance that shows great potential for producing highly conformal doses to treatment volumes.

**Case presentation:**

We present a case of a 67-year-old woman with history of early cervical cancer initially treated by radical laparoscopic hysterectomy. More than 5 years later, the patient presented with a central pelvic vaginal cuff recurrence that is histologically confirmed. Salvage radiotherapy using RapidArc with concurrent cisplatin-based chemotherapy was indicated. A high dose of 70 Gy was delivered to the gross recurrent disease with simultaneous integrated boost (SIB) to the subclinical disease and good sparing of organs at risk especially the rectum and sigmoid.

**Conclusions:**

This case clearly demonstrates a large benefit for salvage RapidArc radiotherapy to central pelvic recurrences of gynecological cancers with an excellent rate of local control and less rate of toxicity.

## Background

Women with early-stage cervical cancer can be potentially cured with radical hysterectomy or chemoradiation therapy [1]. In Morocco, most patients present first at gynecologic clinics and, as a result, the majority of patients of early stage become subjected to radical hysterectomy and lymphadenectomy. Unfortunately, up to 17 % of women develop either local and/or distant disease recurrence usually within the first 2 years of completing the treatment [2, 3].

Local recurrence of cervical cancer after primary surgical therapy is still problematic. It commonly occurs locally as central pelvic recurrences due to the extent of spread into contiguous tissues [4]. Treatment directed to the site of recurrence can be performed with curative intent. Options include radiation therapy (RT) or pelvic exenteration, both resulting in suboptimal rates of local tumor control and rates of survival [5, 6]. Recently, RapidArc radiotherapy is considered an important technological advance that shows great potential for producing highly conformal doses to treatment volumes while sparing organs at risk (OARs).

We report an excellent complete response case of a centrally recurrent squamous cell cervical cancer of the vaginal cuff after radical laparoscopic hysterectomy, successfully managed by salvage RapidArc radiotherapy and concurrent cisplatin-based chemotherapy.

## Case presentation

A 67-year-old female underwent a radical laparoscopic hysterectomy, with adnexectomy and bilateral pelvic lymphadenectomy for a FIGO stage IB1 squamous cell cervical cancer in January 2010. Final pathology report showed a 9-mm squamous cell carcinoma with deep stromal invasion, positive resection margins, and one metastatic lymph node, without vascular embolism or parametrium invasion. Based on pathologic risk factors, adjuvant radiation therapy with radiosensitizing cisplatin was suggested. Unfortunately, the patient was not compliant, she was lost to follow-up and the adjuvant treatment was never delivered.

**Fig. 1:**
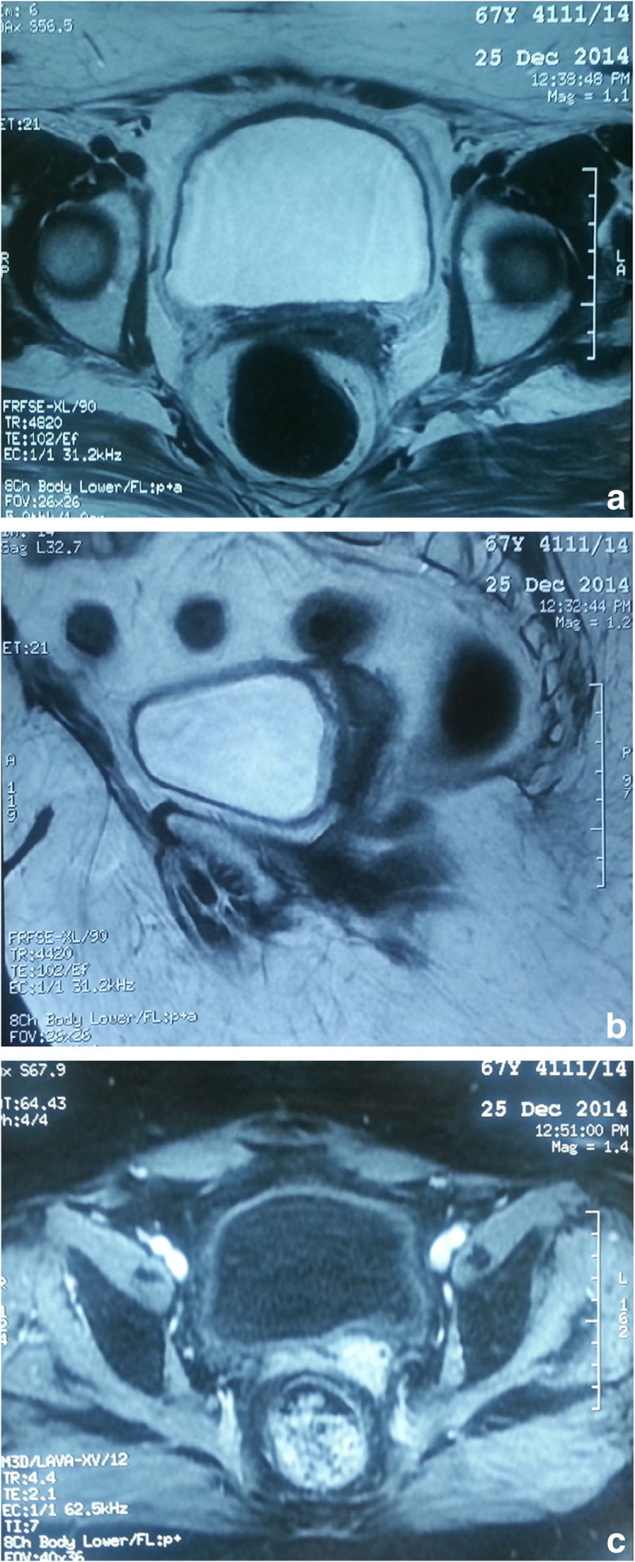
**a**–**c** A pelvis MRI showed a centrally pelvic recurrence of the vaginal cuff. Axial (**a**) and sagittal (**b**) T2W images show intermediate left hemi circumferential signal intensity. Axial (**c**) T1 Gado image shows hyperintense mass in the left cervix

In September 2014, the patient presented to our department with abnormal vaginal bleeding and pelvic pain. On pelvic examination, a tumor of an approximately 4 cm gross circumferential at the vaginal cuff was palpated with left parametrial involvement. A pelvis magnetic resonance imaging (MRI) showed a central pelvic recurrence of the vaginal cuff, as a left hemi circumferential tumor, measuring 44 mm with intimate sigmoid contact and without obstructive uropathy or pelvic wall involvement (Fig. 1). A biopsy of the lesion demonstrated infiltrate and moderately differentiated squamous cell carcinoma of the vaginal cuff. Additional work up including CT scan of the chest and abdomen does not found distant metastasis.

**Fig. 2:**
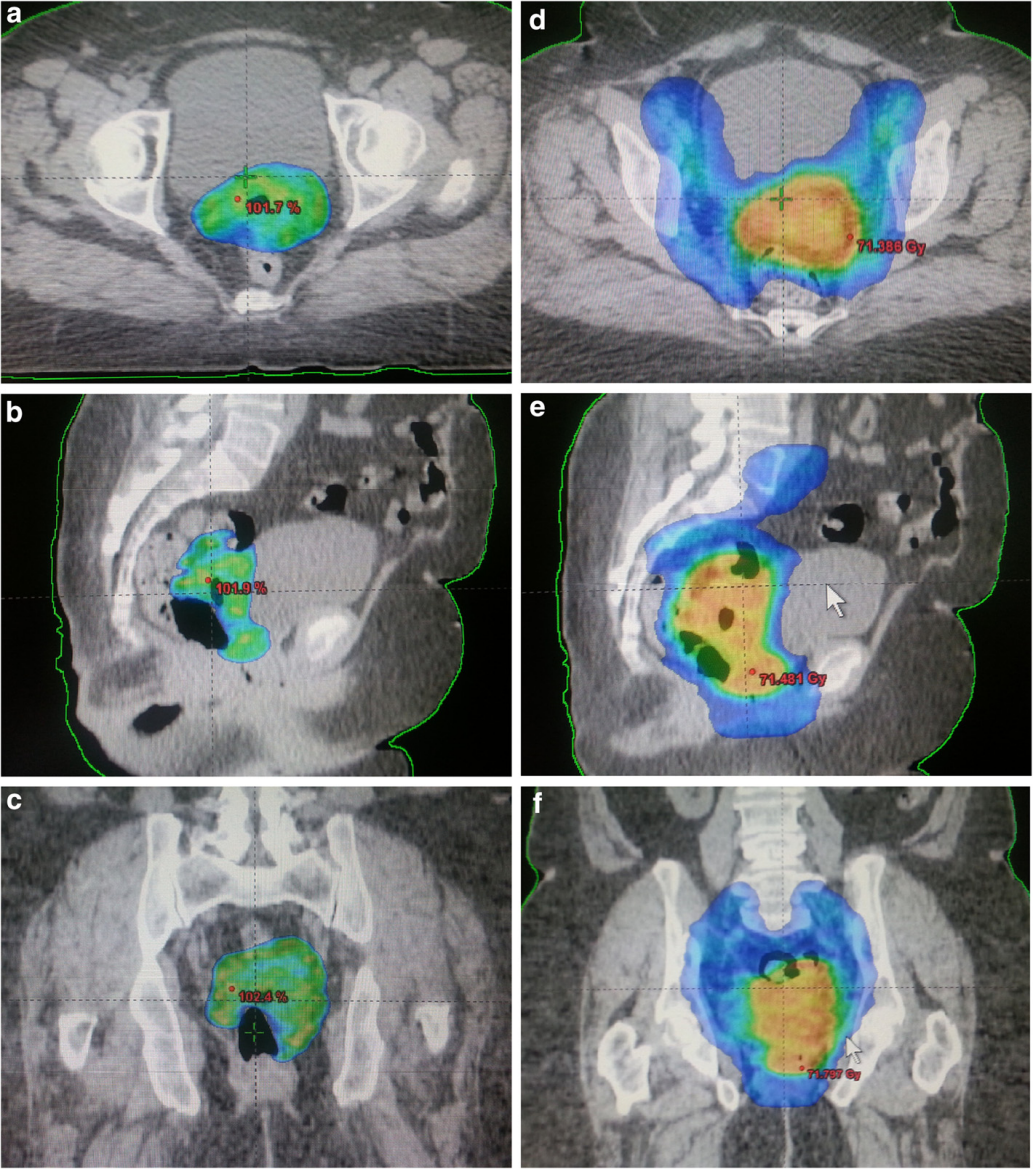
**a**–**f** Axial, sagittal, and coronal images of computed tomography scan dosimetry showed our excellent conformity for dose distribution in radiation therapy

The patient was treated with dual arc RapidArc radiotherapy. The treatment plan was designed to deliver in one process with simultaneous integrated boost (SIB) a dose of 70 Gy to the planning target volume (PTV) based on the gross disease in a 2-Gy daily fraction, 5 days a week. At the same time, the subclinical disease was planned to receive 54 Gy in a 1.5-Gy daily fraction (Fig. 2). Seven cycles of concurrent radiosensitizing cisplatin was also delivered without toxicity.

**Fig. 3:**
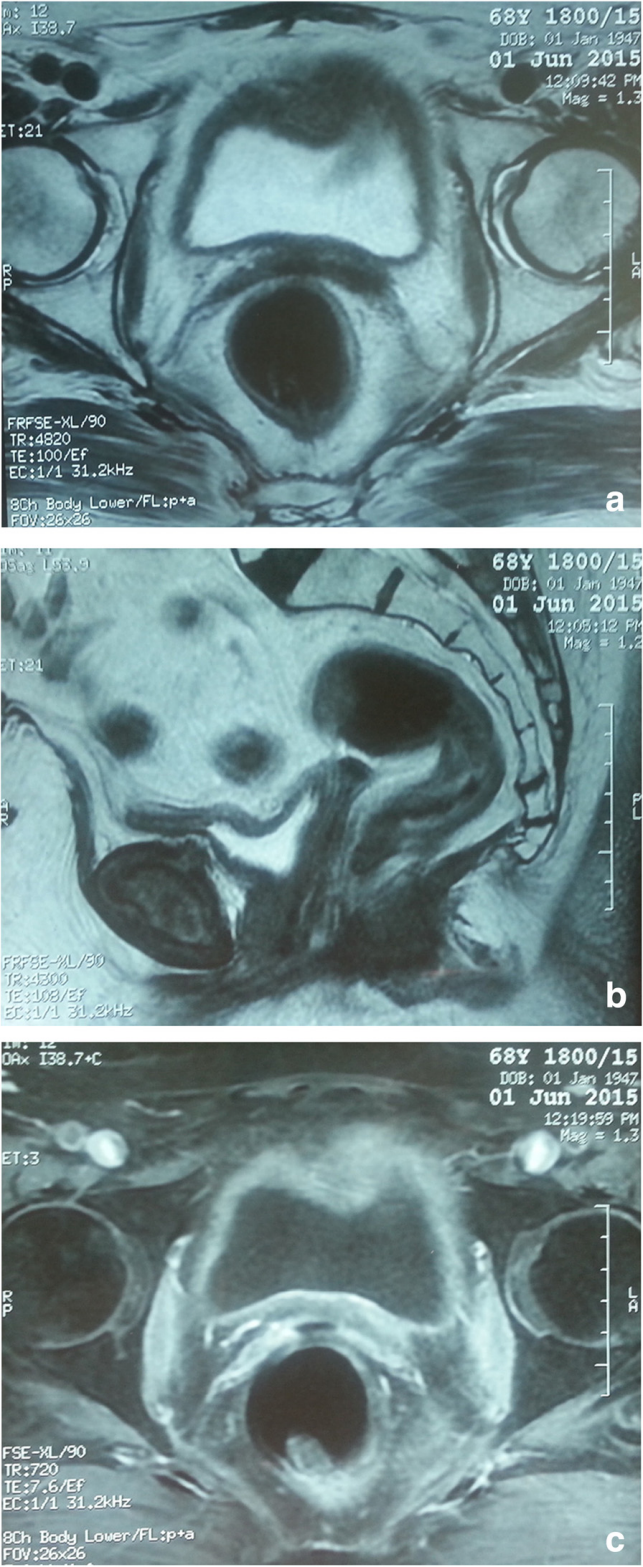
**a**–**c** Axial (**a**, **c**) and sagittal (**b**) MRI images showed a complete response 6 months after completing the treatment

A subsequent clinical evolution was marked by the disappearance of bleeding and the tumor mass. MRI scan realized 6 months after completing treatment showed a complete response (Fig. 3).

### Discussion

After primary surgery for cervical cancer, subsequent pelvic recurrence is effectively managed with external beam radiotherapy and concurrent cisplatin-based chemotherapy due to the presentation of a centrally recurrent cervical cancer of the vaginal cuff, as seen in 35–40 % of cases [7]. Surgical management is an option therapy but it imposes radical pelvic surgery in order to achieve surgical resection with curative intent. However, in locally recurrent disease, radiation offers long-term pelvic control and prolonged survival [8].

One recent retrospective study examining outcomes of patients undergoing salvage radiotherapy alone for recurrent cervical cancer following radical hysterectomy found that is an effective treatment for who recur at the vaginal cuff with relatively little morbidity but it is less effective in patients where recurrence lies outside the central pelvis [9]. Generally, it is accepted that the increasing dose to the target volume in external beam radiotherapy will lead to increased local control. Intensity-modulated radiotherapy (IMRT) is seen as the way to achieve this. It facilitates the delivery of differential doses of radiation to a specified target volume and the escalating of dose prescription. Early experience found that IMRT were able to achieve excellent coverage of target volumes, and there was very little acute gastrointestinal toxicity as compared with conventional external beam radiotherapy techniques [10].

RapidArc radiotherapy is an excellent option used to salvage our patient with a central pelvic vaginal cuff recurrent lesion which seems to obtain better dosimetric results compared to IMRT, with fewer monitor units, and a significant decrease in treatment time [11]. Pelvic radiation can lead to urinary symptoms and bowel changes. However, RapidArc might reduce acute rectal and bladder toxicity compared with conventional techniques [12]. Concurrent cisplatin-based chemoradiotherapy has also been used in the management of recurrent cervical cancer. This approach has provided both better local control and survival with acceptable toxicities in women with locally recurrent cervical cancer in several studies [13, 14].

Surveillance after primary curative therapy for cervical cancer is uniformly recommended, although its effectiveness is not well studied [15]. The concept of long-term surveillance for patients treated with curative intent is based on the premise that early detection of recurrence may lead to treatments that have lower morbidity and increase survival. Early detection of recurrence is aimed at treating patients with potentially curative salvage therapy. This is most likely in patients who have an isolated central pelvic recurrence.

## Conclusions

In summary, we have presented an excellent complete response case of centrally recurrent cervical cancer of the vaginal cuff following radical hysterectomy, which managed by salvage RapidArc radiotherapy with concurrent cisplatin-based chemotherapy. This case demonstrates a large benefit for local dose escalation to the residual tumor by the currently techniques with high rates of local control and less rate of toxicity.

### Consent

Written informed consent was obtained from the patient for publication of this case report and any accompanying images. A copy of the written consent is available for review by the Editor-in-Chief of the journal.
